# Cardiometabolic risk factors in children born with marginally low birth weight: A longitudinal cohort study up to 7 years-of-age

**DOI:** 10.1371/journal.pone.0215866

**Published:** 2019-04-19

**Authors:** Josefine Starnberg, Mikael Norman, Björn Westrup, Magnus Domellöf, Staffan K. Berglund

**Affiliations:** 1 Department of Clinical Sciences, Pediatrics, Umeå University, Umeå, Sweden; 2 Division of Pediatrics, Department of Clinical Science, Intervention and Technology, Karolinska Institutet, Stockholm, Sweden; 3 Division of Neonatology, Department of Women's and Children's Health, Karolinska Institutet, Stockholm, Sweden; 4 Wallenberg Centre for Molecular Medicine (WCMM), Umeå University, Umeå, Sweden; Universidad Miguel Hernandez de Elche, SPAIN

## Abstract

**Introduction:**

Low birth weight (LBW, <2500 g) may predict an increased risk of an adverse cardiometabolic profile later in life, but long-term effects in different populations and birth weight strata are still unclear. We explored laboratory markers of cardiometabolic risk in children born with marginally LBW (2000–2500 g).

**Methods:**

This was a prospective longitudinal cohort study including 285 Swedish marginally LBW children and 95 normal birth weight (NBW, 2501–4500 g) controls. At 3.5 and 7 years of age, blood samples for glucose, insulin, homeostatic model assessment for insulin resistance (HOMA-IR), cholesterol, triglycerides, high- and low density lipoprotein (HDL and LDL), apolipoprotein B (ApoB) and apolipoprotein A1 (ApoA1) were assessed and compared between the groups.

**Results:**

No significant differences in levels of insulin, HOMA-IR, hs-CRP or blood lipids were observed between marginally LBW and NBW children. At 7 years there was a higher proportion of marginally LBW children with elevated levels of insulin, defined as above the 90^th^ percentile of the control group (21% vs 8.6%, p = 0.038). This association was, however, confounded by maternal ethnicity. In marginally LBW children born small for gestational age (SGA), mean fasting glucose was significantly higher compared to controls (4.7 vs 4.5 mmol/L, p = 0.020).

**Conclusions:**

There were no significant differences in insulin, insulin resistance, hs-CRP or blood lipids between the marginally LBW children and controls. The subgroup of marginally LBW children born SGA may present early signs of glucose imbalance already at school age.

## Introduction

Ischemic heart disease is the leading cause of death worldwide and early prevention among those at risk is therefore of great importance [1, 2]. There is an increasing body of evidence suggesting that exposures during early development, for instance pre- and postnatal growth, contribute to adult cardiovascular disease risk [3]. Studies of different cardiometabolic risk factors in children may help to give us a better understanding of both the underlying mechanisms, as well as effects on different long-term outcomes.

Several previous studies have found associations between low birth weight (LBW, <2500 g) and later risk-markers of cardiovascular disease, such as insulin resistance, hypertension, hyperlipidemia and low-grade inflammation [4–8]. However, the effect size of cardiometabolic risk differs with settings, and the mechanisms explaining the associations are partly unclear. LBW children constitute a heterogeneous group with varying gestational ages and with different growth patterns before and after birth; hence their long-term cardiovascular risk may vary.

LBW-children born small for gestational age (SGA) seem to face the largest risk of unfavorable metabolic programming, possibly related to the postnatal accelerated growth [9–11]. The long-term outcome of being born small may relate to several perinatal risk factors such as magnitude of fetal growth restriction, degree of prematurity, postnatal growth acceleration as well as nutritional interventions.

The largest subgroup of LBW children are those born with a birth weight of 2000–2500 g, herein referred to as *marginally LBW* children. This group constitutes 2–15% of all children. Yet, the significance of their future risk of cardiovascular disease is largely unknown [12]. We recently explored growth and body composition in a cohort of 281 marginally LBW children compared to a group of children born with normal birth weight (NBW, 2501–4500 g), and found that the risk of overweight and obesity at 7 years of age did not differ between the two groups. In fact, the marginally LBW children had lower body mass index (BMI) as well as lower fat mass and they were more likely to remain underweight or short compared to NBW children [13, 14]. In the present study, we explored the same cohort regarding their laboratory markers of cardiometabolic risk. Our main objective was to compare marginally LBW and NBW children at 3.5 and 7 years with respect to glucose, insulin, homeostatic model assessment for insulin resistance (HOMA-IR), lipid status, and high sensitive C-reactive protein (hs-CRP). As a secondary aim, we investigated the possible correlation between early iron supplementation as well as early growth and later insulin and glucose levels. Furthermore, we also explored the effects of being small or appropriate for gestational age (SGA or AGA respectively) at birth.

## Methods

### Subjects and study design

This was a prospective, longitudinal cohort study including 285 children born with marginally LBW (2000–2500 g) and 95 control children born at term with NBW (2501–4500 g). The marginally LBW children were recruited by the use of delivery records at two tertiary hospitals in Sweden, Umeå University Hospital, Umeå and Karolinska University Hospital, Stockholm, between March 2004 and November 2007. The subjects were enrolled at 6 weeks of age using the following inclusion criteria: birth weight of 2000–2500 g, no signs of disease at inclusion, no previous blood transfusion and never having received iron supplements.

The included marginally LBW children were originally participants of a double-blinded controlled interventional trial of iron supplements and randomized into three different groups receiving 1 mg iron/kg/day, 2 mg iron/kg/day or placebo between 6 weeks and 6 months of age. The results from the intervention study have been presented elsewhere [15]. In the present follow-up at 7 years, we observed significantly lower blood pressure in children supplemented with iron but no intervention effects on growth were observed at 3.5 years of age [16, 17]. For the laboratory outcomes explored in the present study, no intervention effect was found and we analyzed all three intervention groups together in an observational design.

Prior to the present follow up at 3.5 years of age, every third marginally LBW child was chosen as an index case for matched recruitment of controls. A list was made of the children born closest in time to each index case, with the same sex, at the same hospital, born at term (37–42 weeks) and with NBW. The parents to the first child on the list were contacted and invited to join the study. If they declined the parents to the next child on the list were contacted until each index case had a corresponding control included.

When included in the trial, parents signed a written consent form. This study was approved by regional ethical review boards in Stockholm and Umeå. The funders of the study had no role in study design, data collection, data analysis, data interpretation, or writing of the report. The corresponding author had full access to all the data in the study and had final responsibility for the decision to submit for publication. This study was registered at ClinicalTrials.gov as NCT00558454 (https://clinicaltrials.gov/ct2/show/NCT00558454).

### Data collection

At inclusion (6 weeks of age for LBW children and 3.5 years of age for controls), characteristics such as gestational age and anthropometric measures at birth as well as parental age and education were collected using delivery records and questionnaires. As part of the original iron supplementation study, the LBW children were assessed for weight and length at 6, 12 and 19 weeks and at 6 and 12 months of age. Standard deviation score (SDS) for length and weight were calculated using a sex- and age specific growth standard [18], and SGA was defined as an SDS for birth weight less than -2. All other participants were considered AGA. Presence of intrauterine growth restriction could not be explored, since such prenatal data was not registered.

Included marginally LBW children and controls were invited to follow-ups at 3.5 and 7 years of age. At the first follow-up, parents were asked to fill in a questionnaire regarding family history for non-communicable diseases such as history of diabetes, stroke and hypertension. The parents’ height and weight were assessed at either the 3.5- or the 7-year visit. For the parents who declined measurement or did not come to any of the follow-up visits (20.1% of mothers and 60.3% of fathers), self-reported weight and height were registered. Body mass index (BMI) was calculated (kg body weight/height in m^2^).

Phlebotomy was performed after overnight fasting at both follow-up visits. Before venipuncture, the study participants were offered a local anesthesia patch containing 25 mg Lidocaine and 25 mg Prilocaine. Blood glucose was analyzed directly using a glucose monitor (FreeStyle Mini, Abbott, Alameda, USA) and a 4 ml serum tube was sent to one of the local hospital laboratories at the two participating centers, where it was analyzed for cholesterol, triglycerides, high-density lipoprotein (HDL), low-density lipoprotein (LDL), Apolipoprotein B (ApoB) and Apolipoprotein A1 (ApoA1) using standard, accredited laboratory methods at each study site.

A second serum tube was centrifuged at 3000 rpm for a total of 10 minutes and 0.3 ml of the serum was transferred to micro tubes (Sarstedt, production number 72.730.105) with a pipette and stored in a freezer at -80 degrees. The frozen serum from the 7-year control was analyzed for hs-CRP (R&D Systems, Minneapolis, USA) and for insulin (EMD Millipore, Missouri, USA) using ELISA at the pediatric research laboratory at Umeå University hospital. HOMA-IR was calculated using the formula (insulin [mIU/l] x glucose [mmol/l])/22.5 [19].

Elevated levels of the markers of cardiometabolic risk (subnormal levels for HDL) were defined as cases above the 90^th^ (fasting glucose, insulin, HOMA-IR, triglycerides, cholesterol, LDL, ApoB/ApoA1 and hs-CRP) or below the 10^th^ (HDL) percentiles from the control group.

### Statistical analyses

The power calculation was based on neurocognitive outcomes as presented elsewhere [15, 20].

SPSS 23.0 for Windows (SPSS Inc, Chicago, Illinois) was used for all statistical analyses. As a primary outcome, the mean value of the laboratory cardiometabolic risk markers as well as the prevalence of cases with elevated/subnormal levels, were compared between marginally LBW and controls. Group comparisons were performed using independent student’s t-test when comparing mean values of lipids and glucose since normal distribution could be assumed. Insulin, and consequently HOMA-IR as well as hs-CRP showed a skew distribution including cases below the detection limits. Therefore, these variables were compared using the non-parametric test Mann-Whitney U.

For the dichotomized outcomes, Chi-Square test and Fisher exact test (if n< 5 in any group) were used to assess group differences. Those measurements that differed significantly between the main groups (prevalence of high insulin levels) were further explored using logistic regression models. First, we adjusted for BMI at time of blood sample, since previous studies have shown that BMI associates with insulin. Secondly, we adjusted for the background factor that differed between the groups (maternal birth country).

Finally, in secondary explorative analyses, comparisons were performed between marginally LBW born SGA and controls as well as between marginally LBW born AGA and controls. Furthermore, using linear regression models we also explored the association between iron supplementation as well as early growth rate (ΔSDS in weight) and later glucose and insulin levels in the marginally LBW group. No adjustments for multiple comparisons were applied in these a priori defined analyses.

## Results

**Fig 1:**
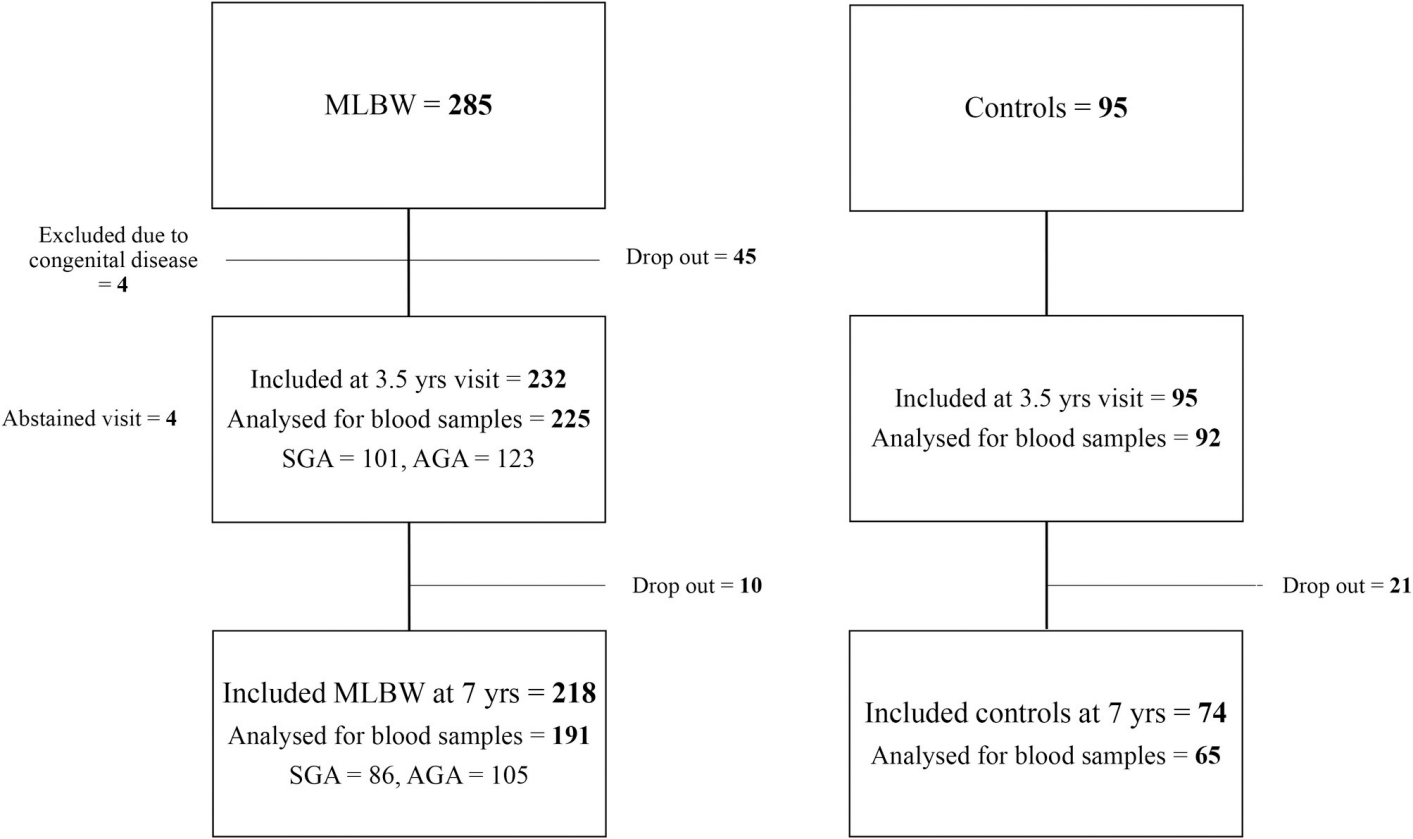
Study flow chart. Study flow chart of 285 marginally low birth weight (marginally LBW) infants and 95 control children born with normal birth weight (NBW). Four infants were excluded due to congenital disorders. Four of the marginally LBW children did not attend the 3.5 year visit but did attend the 7 year visit. The marginally LBW children were also stratified for being born small- or appropriate for gestational age (SGA and AGA respectively).

Of the 285 included marginally LBW children, four were diagnosed with congenital diseases (Williams syndrome, DiGeorge syndrome, Ehler-Danlos syndrome and muscular dystrophy) and excluded in all analyses (Fig 1).

S1 Fig presents the follow-up of the original randomized trial. Of the 232 marginally LBW children and the 95 control children assessed at the 3.5-year visit, successful blood sampling was performed in 225 and 92 children, respectively. At 7 years of age, 218 marginally LBW children and 74 NBW children remained in the study. Of these, blood samples were successfully assessed in 191 marginally LBW and 65 NBW children.

Background and perinatal characteristics of the participating (at 3.5 or 7 years) children are presented in Table 1. Marginally LBW children had a lower prevalence of mothers being born in Scandinavia than controls born at term with NBW, a difference most pronounced among marginally LBW children born SGA. The marginally LBW children also had a greater prevalence of fathers with university education compared to the control group.

Moreover, the marginally LBW children born SGA had slightly older mothers than the marginally LBW children born AGA. Also, a majority of the children born AGA were born preterm, while most of the children born SGA were born at term. Regarding early growth, 44% of included marginally LBW children had a growth in weight of >1SDS during the first year of life, with a majority being born SGA. More detailed data of early growth has been published elsewhere [14]. None of the children was treated with growth hormone.

The marginally LBW children that dropped out before the 3.5 year visit had higher Apgar score at 5 minutes (9.7 vs 9.,5 p = 0.035) compared to those participating at 3.5 year. In addition there was a higher prevalence of drop out from the study centre in Stockholm compared to Umeå (20% vs 1% of the participants in Stockholm and Umeå respectively, p = 0.006).

**Table 1:** Cohort characteristics of marginally low birth weight (marginally LBW) children, including stratification for small- and appropriate for gestational age (SGA and AGA), and control children with normal birth weight.

		Marginally LBW		
	Controls	All marginally LBW	AGA	SGA
	*N = 95*	*N = 236*	*N = 131*	*N = 105*
**Pregnancy**				
Maternal smoking during pregnancy	3 (3.2%)	7 (3.0%)	2 (1.6%)	5 (4.9%)
**Infant**				
Female gender	48 (51%)	121 (52%)	73 (56%)	48 (46%)
Gestational age at birth (wks)	40.0 (1.2)	36.5 (1.9)	35.2 (1.4)	38.0 (1.1)
Born preterm (< 37 wks)	0 (0.0%)	131 (52%)	114 (87%)	17 (16%)
Birth weight (kg)	3.56 (0.4)	2.29 (0.1)	2.30 (0.2)	2.28 (0.1)
Birth length (cm)	50.6 (2.0)	45.3 (1.4)	45.0 (1.4)	45.7 (1.3)
Growth in weight first 12 mos >1 SDS	ND	101 (44%)	35 (28%)	66 (64%)
**Parental characteristics**				
Mother born in Scandinavia	69 (93%)	197 (84%)*	115 ((89%)	82 (79%)*
Mother's age at birth of child (yrs)	31.7 (4.6)	31.8 (5.0)	31.2 (4.6)	32.5 (5.4)
Mother's BMI (kg/m^2^)	23.4 (3.8)	24.0 (4.9)	23.7 (4.5)	24.4 (5.4)
Father's BMI (kg/m^2^)	25.5 (3.3)	25.6 (3.3)	25.7 (3.4)	25.3 (3.1)
Mother educated at university	58 (61%)	129 (55%)	68 (52%)	61 (59%)
Father educated at university	45 (47%)	140 (60%)*	79 (61%)*	61 (59%)
**Family history†**				
Hypertension	49 (52%)	140 (61%)	63 (62%)	77 (60%)
Diabetes	26 (27%)	65 (28%)	34 (33%)	31 (24%)
Stroke	15 (16%)	41 (18%)	14 (14%)	27 (21%)
Cardiovascular disease	23 (24%)	63 (27%)	32 (31%)	31 (24%)

Data are mean (SD) or No (%).

*p-value <0.05 compared to controls using Chi-square test for categorical variables and independent T-test for continuous variables.

†First or second degree relative to any parent. ND is no data available.

The results from the measures of the laboratory markers of cardiometabolic risk at 3.5 and 7 years of age are presented in Tables 2 and 3, respectively. There were no overall differences between marginally LBW children and controls in any of the laboratory outcomes when analyzed as continuous variables. Excluding cases of CRP > 3 mg/L did not change the results.

**Table 2:** Laboratory metabolic markers at 3.5 years of age in 225 children born with marginally low birth weight (marginally LBW) compared to 92 healthy control children born at term with normal birth weight. Including stratified analyses for small- and appropriate for gestational age (SGA and AGA).

		Marginally LBW		
	Controls	All marginally LBW	AGA	SGA
* *	*n = 92*	*n = 225*	*n = 123*	*n = 101*
Fasting glucose (mmol/L)	5.0 (0.6)	5.0 (0.6)	5.0 (0.5)	5.0 (0.6)
Cholesterol level (mmol/L)	4.1 (0.7)	4.2 (0.7)	4.2 (0.7)	4.3 (0.8)
Triglyceride level (mmol/L)	0.82 (0.4)	0.95 (0.5)	0.95 (0.4)*	0.94 (0.6)
HDL (mmol/L)	1.2 (0.3)	1.2 (0.3)	1.2 (0.3)	1.2 (0.3)
LDL (mmol/L)	2.6 (0.7)	2.6 (0.6)	2.6 (0.6)	2.6 (0.7)
Apo B (g/L)	0.83 (0.2)	0.84 (0.2)	0.84 (0.2)	0.83 (0.2)
Apo A1 (g/L)	1.3 (0.2)	1.3 (0.2)	1.3 (0.2)	1.3 (0.2)
ApoB/ApoA1	0.66 (0.2)	0.65 (0.2)	0.65 (0.2)	0.64 (0.2)

Data are mean (SD), n are numbers of included participants.

*p-value is <0.05 compared to controls using independent t-test.

**Table 3:** Laboratory metabolic markers at 7 years of age in 191 children born with marginally low birth weight (marginally LBW) compared to 65 healthy controls born term with normal birth weight. Including stratified analyses for small- and appropriate for gestational age (SGA and AGA).

		Marginally LBW		
	Controls	All marginally LBW	AGA	SGA
* *	*n = 65*	*n = 191*	*n = 105*	*n = 86*
Fasting glucose (mmol/L)	4.5 (0.5)	4.6 (0.5)	4.5 (0.5)	4.7 (0.5)*
Fasting insulin level (μU/mL)	2.8 (<0.2–3.5)	2.7 (2.3–3.8)	2.6 (2.1–3.7)	2.9 (2.2–4.1)
HOMA-IR	0.60 (<0.2–0.7)	0.56 (0.4–0.8)	0.55 (0.4–0.8)	0.62 (0.4–0.8)
Cholesterol level (mmol/L)	4.5 (0.8)	4.4 (0.7)	4.4 (0.8)	4.4 (0.7)
Triglyceride level (mmol/L)	0.57 (0.2)	0.60 (0.2)	0.61 (0.3)	0.58 (0.2)
HDL (mmol/L)	1.4 (0.3)	1.5 (0.4)	1.4 (0.3)	1.5 (0.4)
LDL (mmol/L)	2.8 (0.7)	2.7 (0.6)	2.7 (0.6)	2.6 (0.7)
Apo B (g/L)	0.81 (0.2)	0.82 (0.2)	0.84 (0.2)	0.80 (0.2)
Apo A1 (g/L)	1.4 (0.2)	1.4 (0.3)	1.4 (0.2)	1.5 (0.3)
ApoB/ApoA1	0.57 (0.1)	0.61 (0.3)	0.64 (0.3)	0.58 (0.3)
hs-CRP (mg/L)	0.19 (0.10–0.48)	0.23 (0.12–0.67)	0.24 (0.13–0.83)	0.22 (0.12–0.65)

Data are mean, SD or median (IQR). N are numbers of included participants.

*p-value <0.05 compared to controls using independent t-test or Mann-Whitney U test.

Lowest detection limit was 0.2 μU/L for insulin and 0.078 mg/L for CRP.

**Fig 2:**
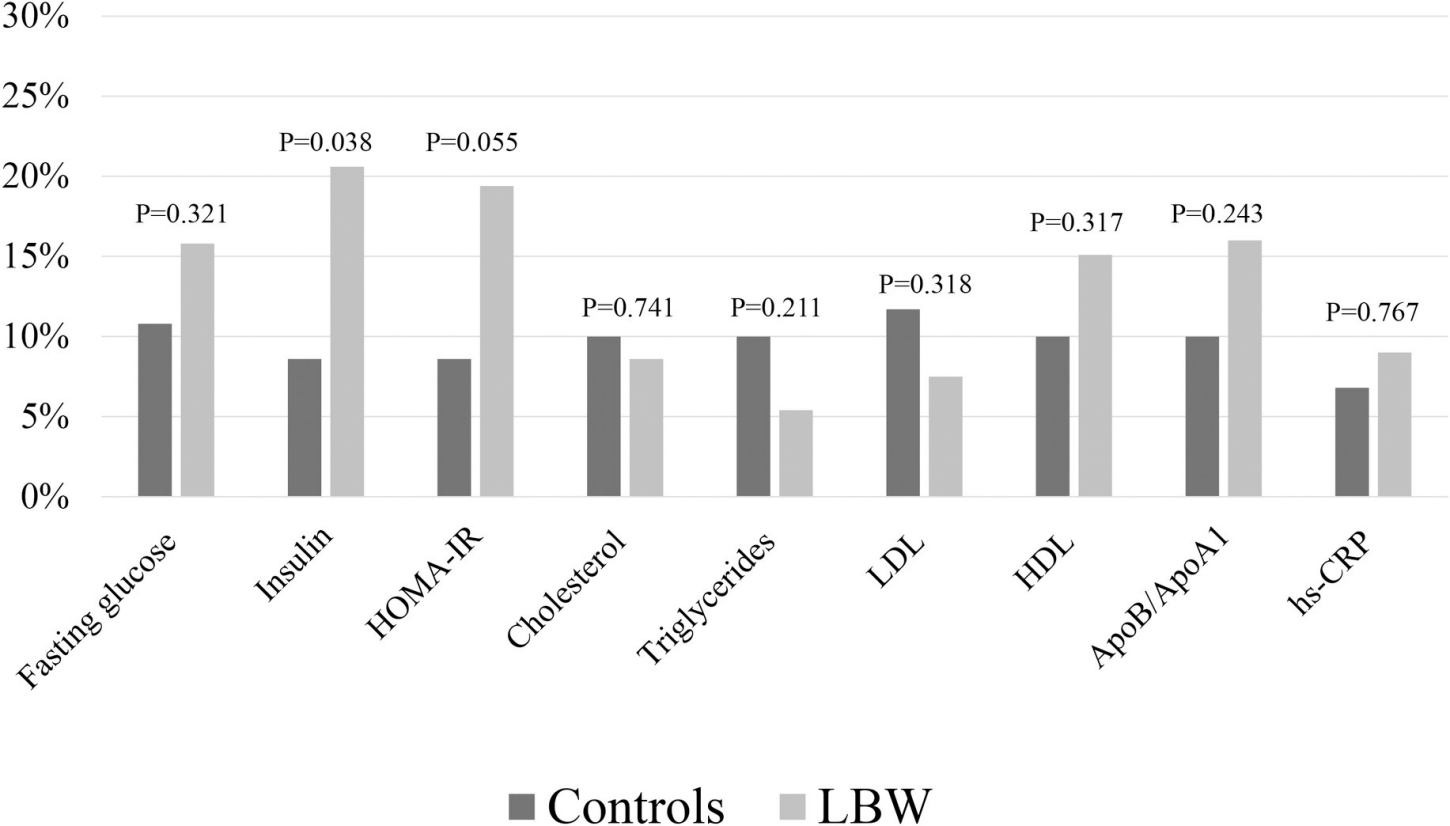
Prevalence of elevated cardiometabolic markers in marginally LBW children compared to controls. The prevalence of children with elevated markers of cardiometabolic risk (subnormal levels for HDL) at 7 years of age in 191 marginally low birth weight (marginally LBW) compared to 65 control children born with normal birth weight. P-values are Chi-square test for group comparison.

The prevalence of children with elevated/subnormal levels of the laboratory markers at 7 years of age are presented in Fig 2. For insulin, there was a significantly higher proportion of cases with elevated levels in the marginally LBW children compared to the controls. A logistic regression model showed that the odds ratio (OR) for elevated levels compared to control children were 2.74 (95% CI: 1.02–7.35), p = 0.045. When adding BMI as a possible confounder in the model, the effect size further increased (3.13 [95% CI: 1.15–8.52], p = 0.025). However, when also adding whether the mother was born in Scandinavia or not, the OR was reduced to 2.41 (95% CI: 0.88–6.66), p = 0.089, suggesting a confounding effect from ethnicity.

The secondary analyses stratified for being born AGA and SGA further showed that the AGA subgroup of marginally LBW children had significantly higher levels of triglycerides than the controls (0.95 vs 0.82 mmol/L, p = 0.043) at 3.5 years of age (Table 2). Moreover, at 7 years mean fasting glucose was significantly higher in the SGA subgroup of marginally LBW children compared to the control group (4.7 vs 4.5 mmol/L, p = 0.020) (Table 3). This group difference was not confounded by BMI of the child or maternal birth country.

The correlation between early iron supplementation as well as early growth rate (ΔSDS) in weight with later levels of glucose and insulin is presented in S1 Table. Iron supplementation did not affect glucose and insulin levels at 7 years of age. Regarding growth, there was a positive correlation between growth in weight at 0–6 weeks of age and later glucose levels (p = 0.028), but no correlation to levels of insulin.

## Discussion

In the present study, we explored the largest group of LBW children (2000–2500 g) and used several validated laboratory markers considered important for the future risk of developing metabolic syndrome, diabetes and cardiovascular disease. While we in our previous studies of overweight and body composition in these children, failed to show any increased risk in the marginally LBW cohort [13], the present may indicate that the children born with marginally LBW, and especially those born SGA, have signs of altered insulin and glucose homeostasis, already at 7 years of age.

In dichotomized analyses, the marginally LBW children had a higher prevalence of elevated levels of insulin. However, while BMI enhanced the association, having a non-Scandinavian mother was a confounder that reduced the OR below the level of significance, although still with a notable effect size (OR: 2.41). The correlation to maternal birth country has been reported previously by Bennet et al, who showed an independent correlation between ethnicity and insulin sensitivity [21]. Altogether, despite the unclear clinical relevance of this finding, it still emphasizes the importance of genetic traits when exploring cardiovascular risk and encourage further research within the area. In the present study, we did not find any significant correlation between early growth rate and insulin levels in the marginally LBW group, even though the sample size limited these secondary analyses.

Our observations with regard to imbalanced glucose metabolism are in concordance with several previous reports of other subgroups of LBW children, suggesting that LBW may relate to insulin resistance later in life [7, 22]. Mostly, studies of children born even smaller or those born with distinct growth restriction, seem to be at largest risk. Kajantie et al reported increased risk of insulin resistance and glucose intolerance at 25 years of age in adults born with very low birth weight (<1500 g) [23]. Other studies have suggested that mainly children born SGA, and especially those with postnatal catch-up growth, are more sensitive to insulin imbalance than those born AGA [9, 10, 24]. This could also be applicable for marginally LBW children born SGA, who did present the most rapid early weight gain in this cohort [14]. This is further supported by our observation that weight gain from birth to 6 weeks of age correlated positively to later fasting glucose in marginally LBW children.

The mechanisms behind early programming of later insulin resistance and glucose intolerance are partly unknown. It has been hypothesized that the low weight fetus or infant adapts to intrauterine and early life conditions, adaptions that eventually lead to an increased cardiovascular risk later in life. As recently reviewed by Martin-Gronert and Ozanne, both animal and human studies have shown a reduced number of glucose transporters (GLUT4) in muscle and adipose tissue, following intrauterine growth restriction. As a consequence, glucose uptake is compromised, leading to elevated plasma glucose, as observed in the children born SGA. Also, it has been suggested that there is a critical window in development of beta cells in pancreas during fetal and early postnatal life. Children who experience early growth restriction have a reduced number of beta-cells, leading to difficulties in compensating an increased demand of insulin secretion, also resulting in elevated plasma glucose. These alterations have been suggested as the initial targets in metabolic programming [25]. Since we did not observe any significant difference in HOMA-IR, a marker for insulin resistance, a deficient insulin secretion could explain the high fasting glucose in those born SGA.

With regard to lipid status and hs-CRP, our study did not show any differences in continuous or dichotomized outcomes between marginally LBW children and control children. This could imply that the risk for adverse lipid profile and low grade inflammation is low or absent in this group of children. It might also suggest that the analyses were made at a too low age and that such differences in cardiometabolic risk profile between the groups might emerge later, in concordance to previously published observational studies [6, 26–28]. Moreover, even though the main analyses of blood lipids did not show any increased risk in marginally LBW born children, we did observe that marginally LBW children born AGA had higher levels of triglycerides than the control children at 3.5 years. Since there were no similar results in the other lipid status markers at any of the follow-up visits and the difference was not seen at 7 years, the result may represent a type I error. However, this finding may also suggest that marginally LBW due to preterm birth (87% of the marginally LBW born AGA were born preterm), is a risk factor for long-term alteration in lipid profile. According to two reviews, there is limited evidence of a correlation between birth weight and later triglycerides in children, even though it has been established in retrospective studies and animal trials. Results have been inconsistent and the clinical relevance is still unclear [5, 6, 29, 30]. Further studies regarding the effect of LBW on lipid status are warranted.

The strengths of this study include its prospective and longitudinal design of a fairly large cohort of children with marginally LBW. Furthermore, the follow-up assessments were comprehensive, including blood sampling at both 3.5 and 7 years-of-age, and included several important and clinically relevant outcomes. The study was limited by its observational design where causality cannot be determined. Compared to several previous retrospective register studies, our sample size was also limited to detect associations of lower magnitude. Furthermore, the cohort of Swedish marginally LBW children was rather heterogeneous, including different degrees of prematurity and growth restriction, and also most likely included both cases of intrauterine growth restriction and other causes–such as constitutional factors–to being born SGA. We cannot determine to what extent our findings would be representative for another setting. Nevertheless, marginally LBW subjects represent a large subgroup of infants, children and adults, which so far has been given limited focus in research and in daily clinical practice, and we believe that the present study adds important knowledge with regards to their risk in the global epidemic of cardiovascular disease, diabetes and metabolic syndrome.

## Conclusion

Reassuringly, we found no evidence of increased cardiometabolic risk in the overall marginally LBW group up to 7 years of age. However, the subgroup of those born SGA did present with a higher fasting glucose, which could be an early indicator for later type 2 diabetes mellitus. Further studies of larger size reaching beyond childhood is warranted to determine the magnitude of this possible cardiometabolic risk, its clinical relevance, and the underlying mechanism.

## Supporting information

S1 Fig Flow chart of the randomization follow-up at 3.5 and 7 years of age. Flow chart of the 95 included normal birth weight controls (2501–4500 g) and 285 included marginally low birth weight children (2000-2500g) randomized to receive placebo or iron (Fe) supplementation during 6 weeks and 6 months of age. *When analyzing the effect of the iron intervention, 16 children were excluded at 6 weeks due to diagnosed anemia (Hb<90g/L) and 2 due to blood disorder. **Nine infants were prescribed iron from 12 weeks of age due to suspected iron deficiency anemia but these were included in the analyses of iron intervention according to an intention to treat principle.
(TIF)

S1 Table Association between iron intervention as well as early weight gain with glucose and insulin at 7 years of age. (DOCX)
