# Supplementary figures and images for: Cardiometabolic risk factors in children born with marginally low birth weight: A longitudinal cohort study up to 7 years-of-age
Source: PLoS One. 2019 Apr 19;14(4):e0215866. doi: 10.1371/journal.pone.0215866 (PMC6474616; doi:10.1371/journal.pone.0215866)

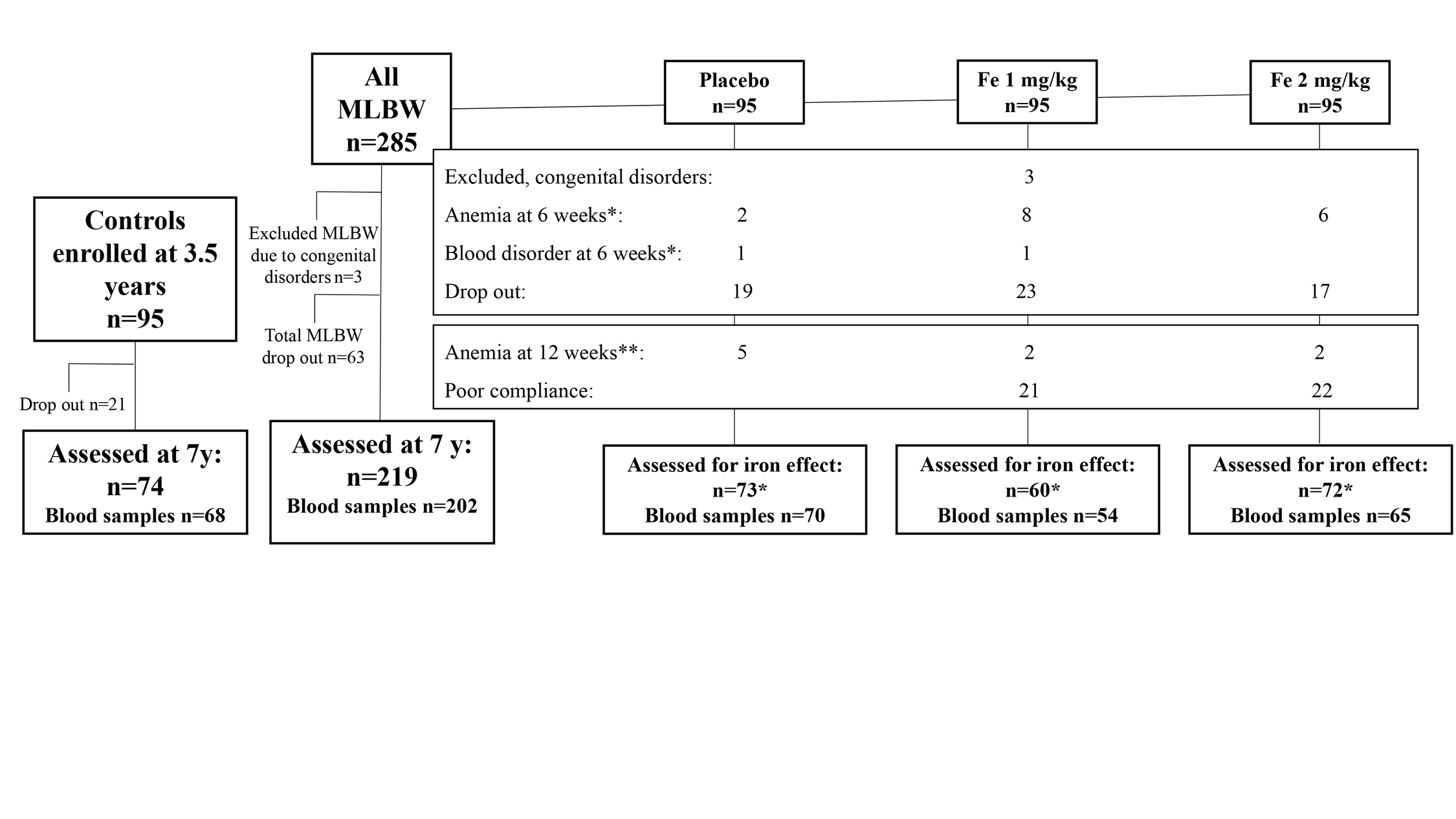

Supplement: S1 Fig — Flow chart of the 95 included normal birth weight controls (2501–4500 g) and 285 included marginally low birth weight children (2000-2500g) randomized to receive placebo or iron (Fe) supplementation during 6 weeks and 6 months of age. *When analyzing the effect of the iron intervention, 16 children were excluded at 6 weeks due to diagnosed anemia (Hb<90g/L) and 2 due to blood disorder. **Nine infants were prescribed iron from 12 weeks of age due to suspected iron deficiency anemia but these were included in the analyses of iron intervention according to an intention to treat principle. (TIF) [file pone.0215866.s001.tif]
