# Supplementary material for: Cardiometabolic risk factors in children born with marginally low birth weight: A longitudinal cohort study up to 7 years-of-age
Source: PLoS One. 2019 Apr 19;14(4):e0215866. doi: 10.1371/journal.pone.0215866 (PMC6474616; doi:10.1371/journal.pone.0215866)
Supplement: S1 Table — (DOCX) [file pone.0215866.s002.docx]

|  | **Glucose (mmol/L)** | | | | **Insulin (µU/mL)** | | |
| --- | --- | --- | --- | --- | --- | --- | --- |
|  | **B (95% CI)** | **r^2^** | **p** | **B (95% CI)** | | **r^2^** | **p** |
| *Iron intervention* |  |  |  |  | |  |  |
| Iron supplementation 6 wks-6 mos | -0.015 (-0.17; 0.14) | -0.005 | 0.849 | 0.056 (-0.54; 0.66) | | -0.005 | 0.855 |
|  |  |  |  |  | |  |  |
| *Early growth in weight (*∆*SDS)* |  |  |  |  | |  |  |
| 0-6 wks | 0.089 (0.01; 0.17) | 0.020 | 0.028 | 0.143 (-0.17; 0.46) | | -0.001 | 0.373 |
| 6-12 wks | 0.059 (-0.07; 0.19) | -0.001 | 0.893 | 0.037 (-0.47; 0.55) | | -0.006 | 0.886 |
| 12-19 wks | 0.130 (-0.03; 0.29) | 0.008 | 0.116 | -0.026 (-0.65; 0.60) | | -0.006 | 0.933 |
| 19-6 mos | 0.107 (-0.08; 0.30) | 0.001 | 0.107 | -0.096 (-0.82; 0.63) | | -0.005 | 0.796 |
| 6-12 mos | -0.007 (-0.13; 0.11) | -0.005 | 0.906 | 0.088 (-0.39; 0.56) | | -0.005 | 0.714 |

B is unstandardized regression coefficient with 95% CI using linear regression and r is standardized coefficient.
